# Supplementary material for: Gene2vec: distributed representation of genes based on co-expression
Source: BMC Genomics. 2019 Feb 4;20(Suppl 1):82. doi: 10.1186/s12864-018-5370-x (PMC6360648; doi:10.1186/s12864-018-5370-x)
Supplement: Supplementary file 1 — Supplementary figures (Figure S1 to Figure S27). (DOCX 13027 kb) [file 12864_2018_5370_MOESM1_ESM.docx]

**
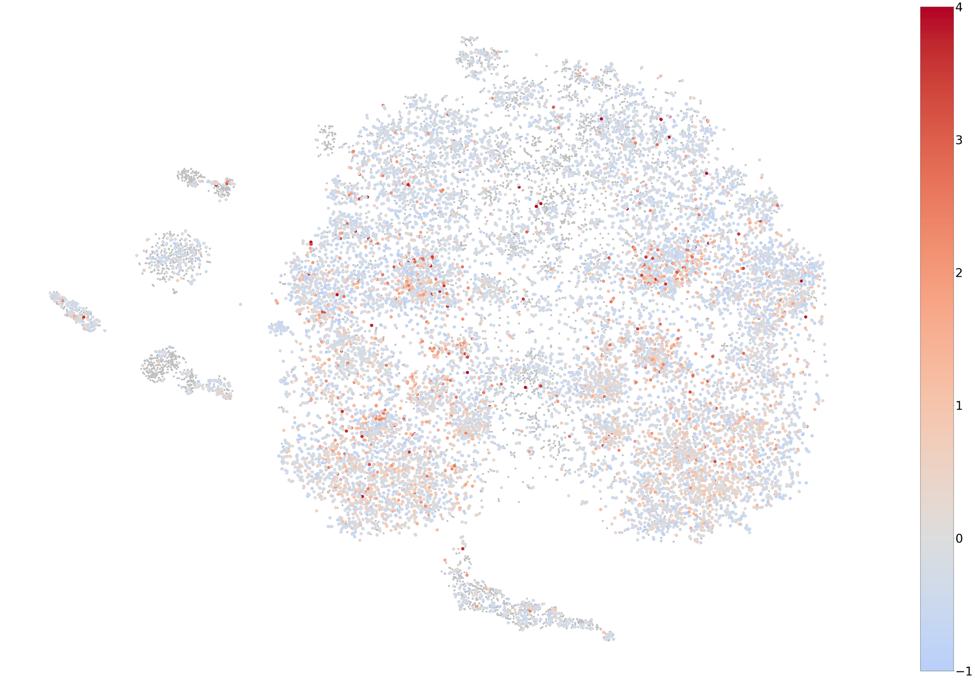
**

Adipose

**Figure S1**: Gene co-expression map with genes colored by z-scores obtained using GTEx adipose expression data.


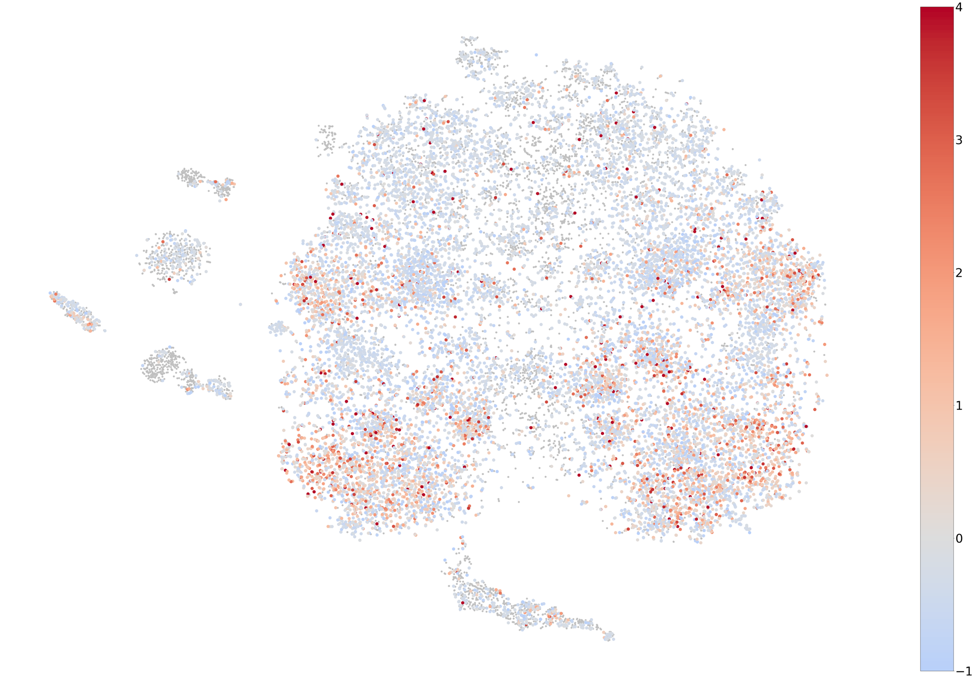


Adrenal gland

**Figure S2**: Gene co-expression map with genes colored by z-scores obtained using GTEx adrenal gland expression data.


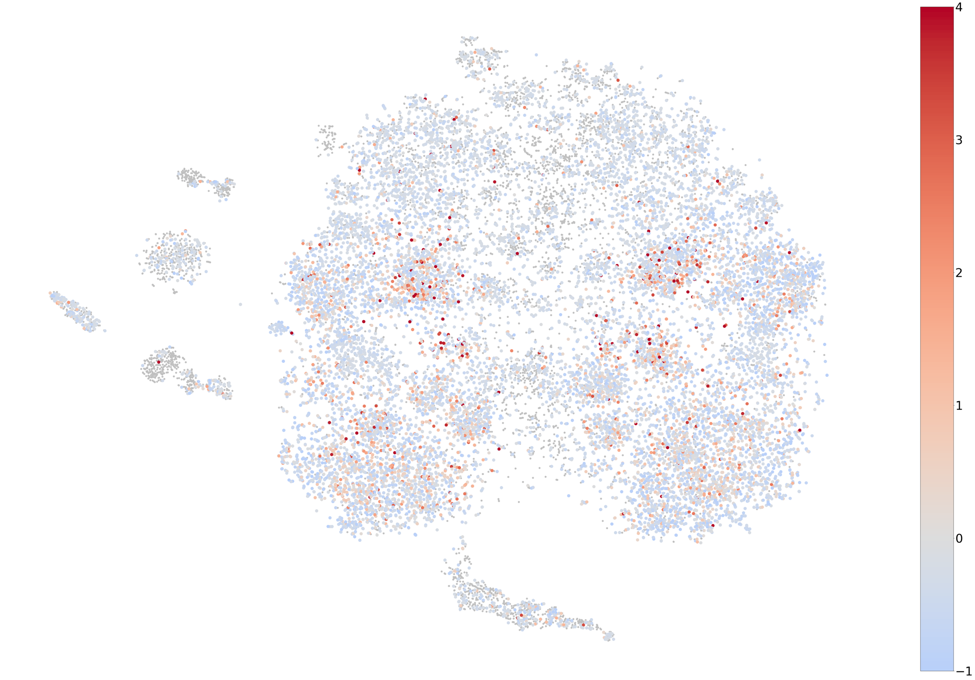


Blood vessel

**Figure S3**: Gene co-expression map with genes colored by z-scores obtained using GTEx blood vessel expression data.


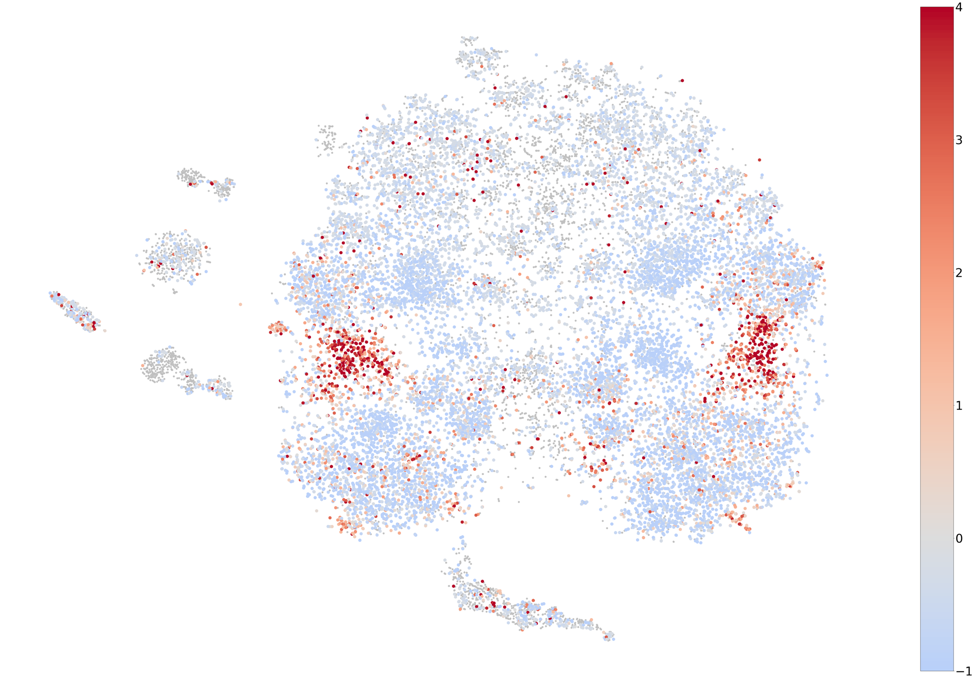


Blood

**Figure S4**: Gene co-expression map with genes colored by z-scores obtained using GTEx blood expression data.


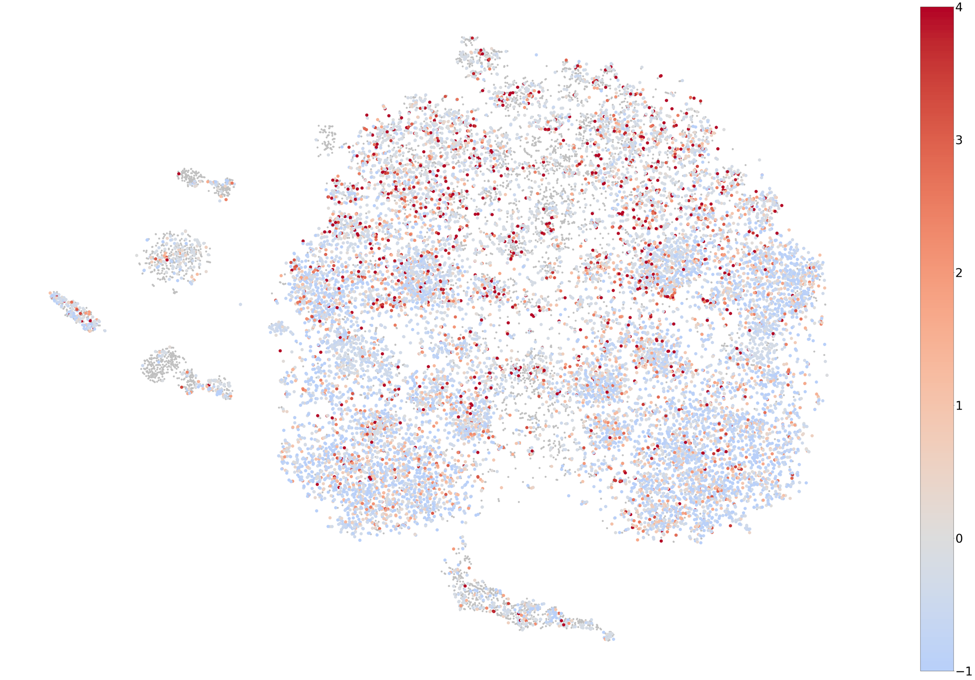


Brain

**Figure S5**: Gene co-expression map with genes colored by z-scores obtained using GTEx brain expression data.


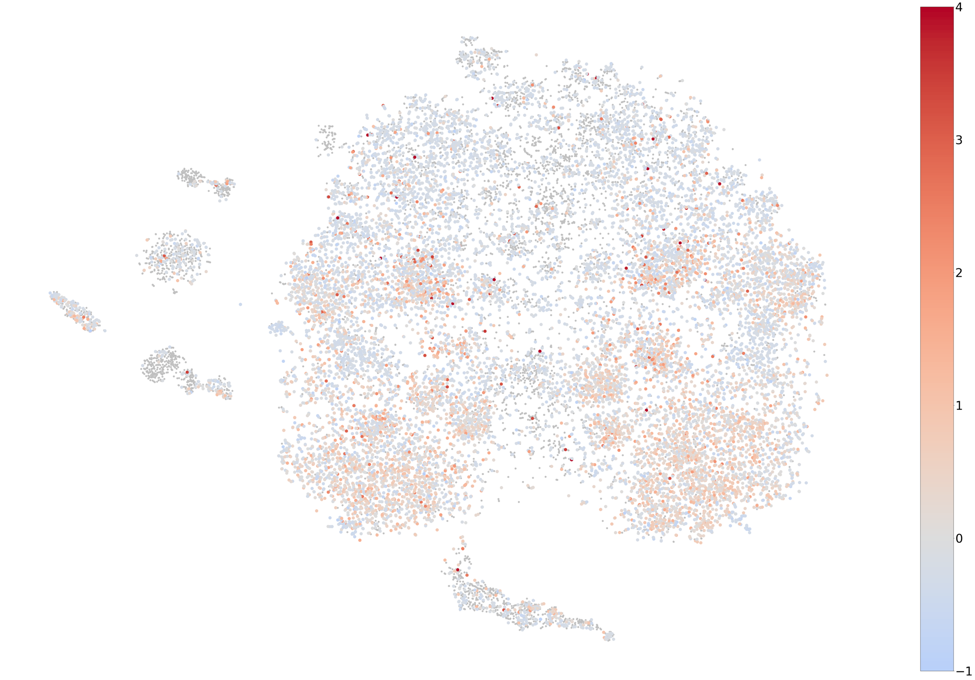


Breast

**Figure S6**: Gene co-expression map with genes colored by z-scores obtained using GTEx breast expression data.


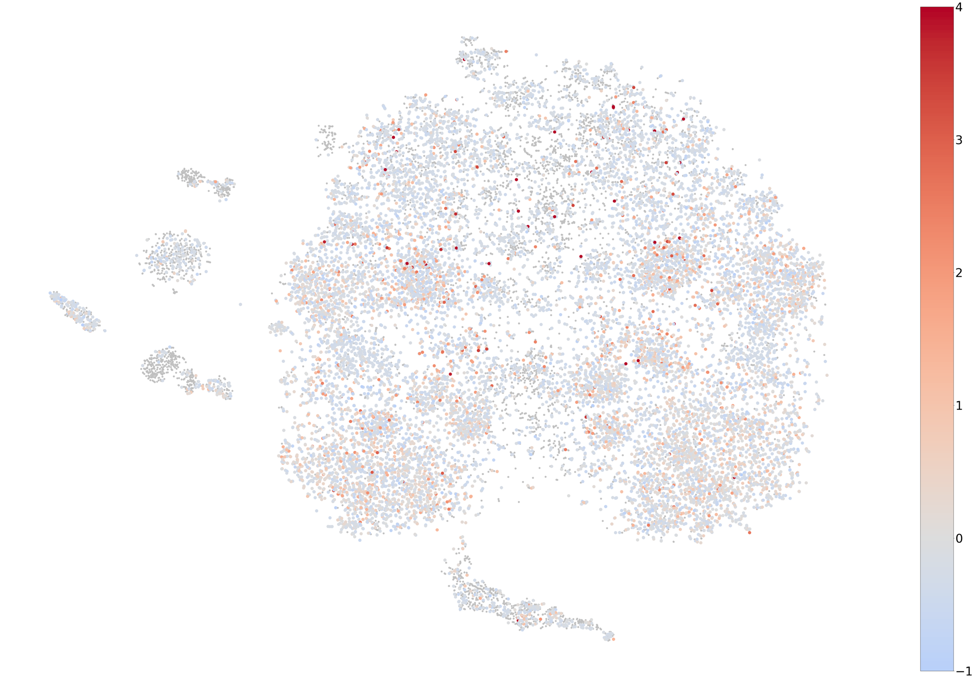


Colon

**Figure S7**: Gene co-expression map with genes colored by z-scores obtained using GTEx colon expression data.


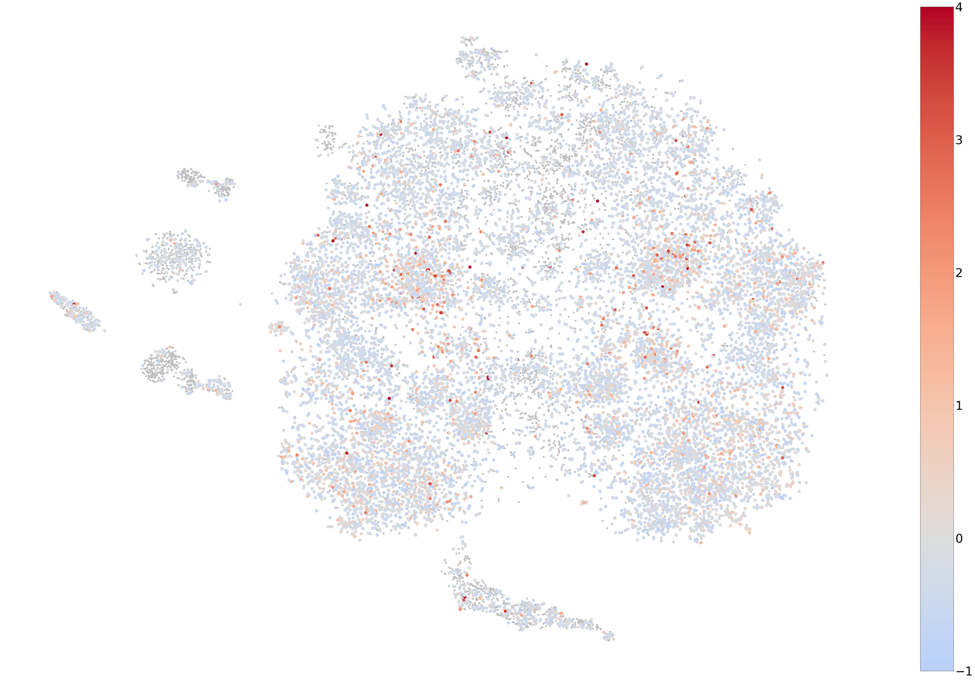


Esophagus

**Figure S8**: Gene co-expression map with genes colored by z-scores obtained using GTEx esophagus expression data.


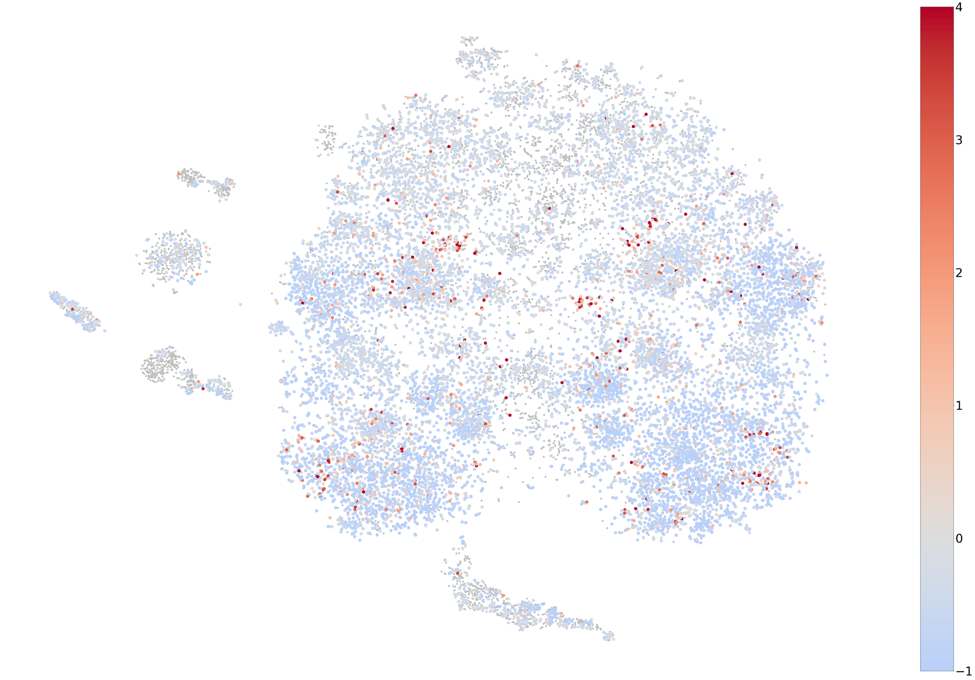


Heart

**Figure S9**: Gene co-expression map with genes colored by z-scores obtained using GTEx heart expression data.


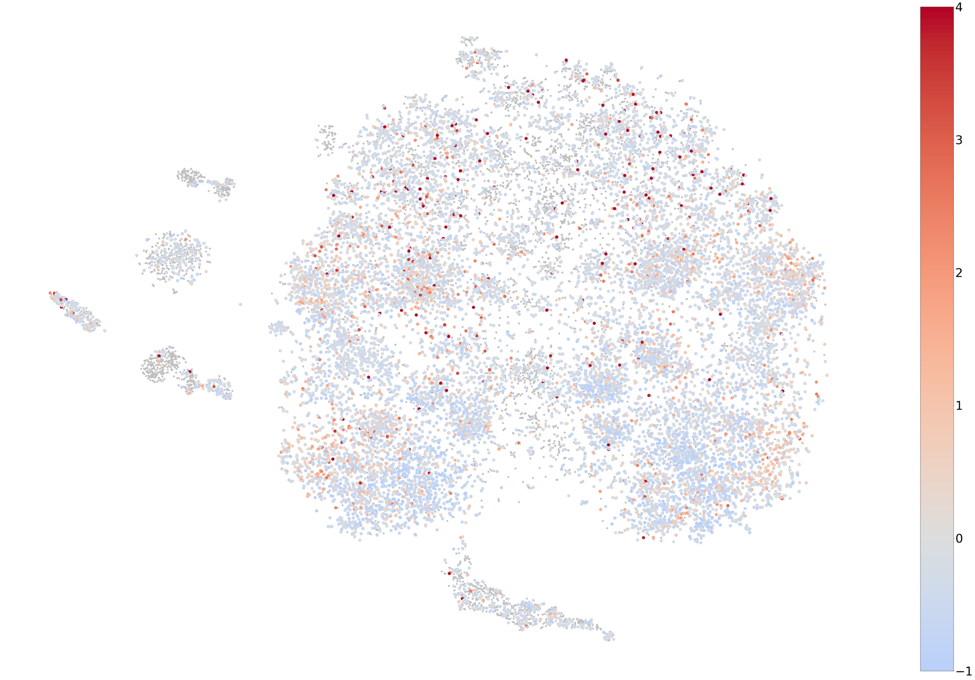


Kidney

**Figure S10**: Gene co-expression map with genes colored by z-scores obtained using GTEx kidney expression data.


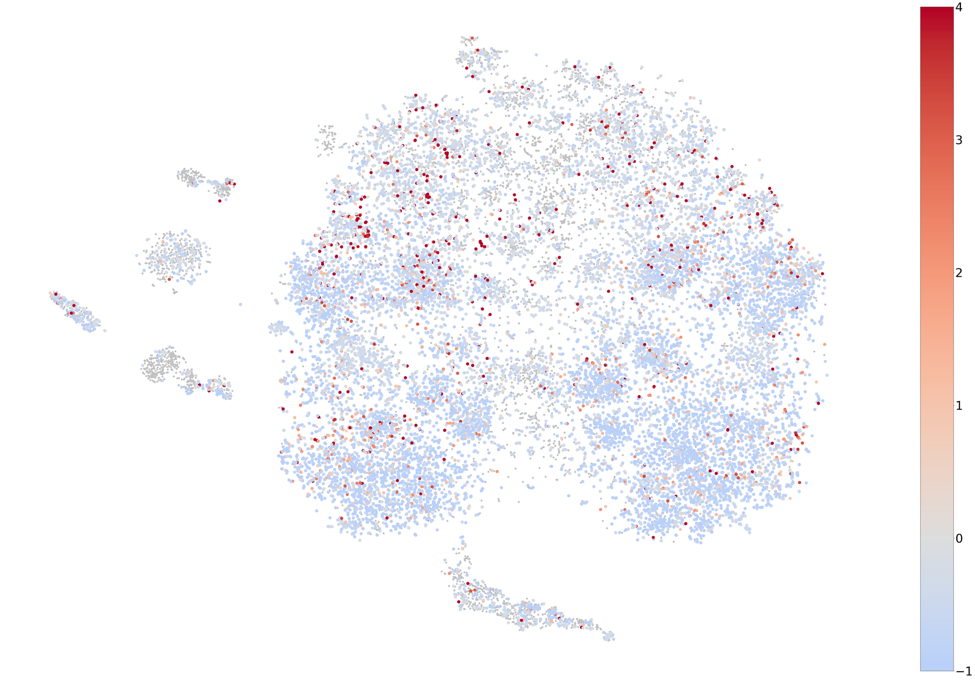


Liver

**Figure S11**: Gene co-expression map with genes colored by z-scores obtained using GTEx liver expression data.


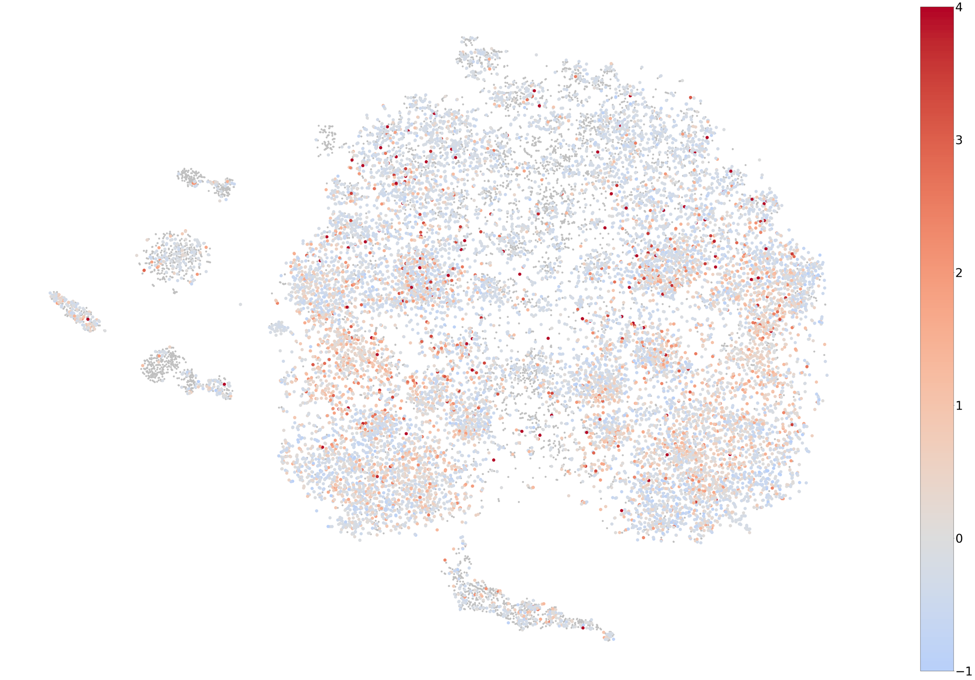


Lung

**Figure S12**: Gene co-expression map with genes colored by z-scores obtained using GTEx lung expression data.

Muscle


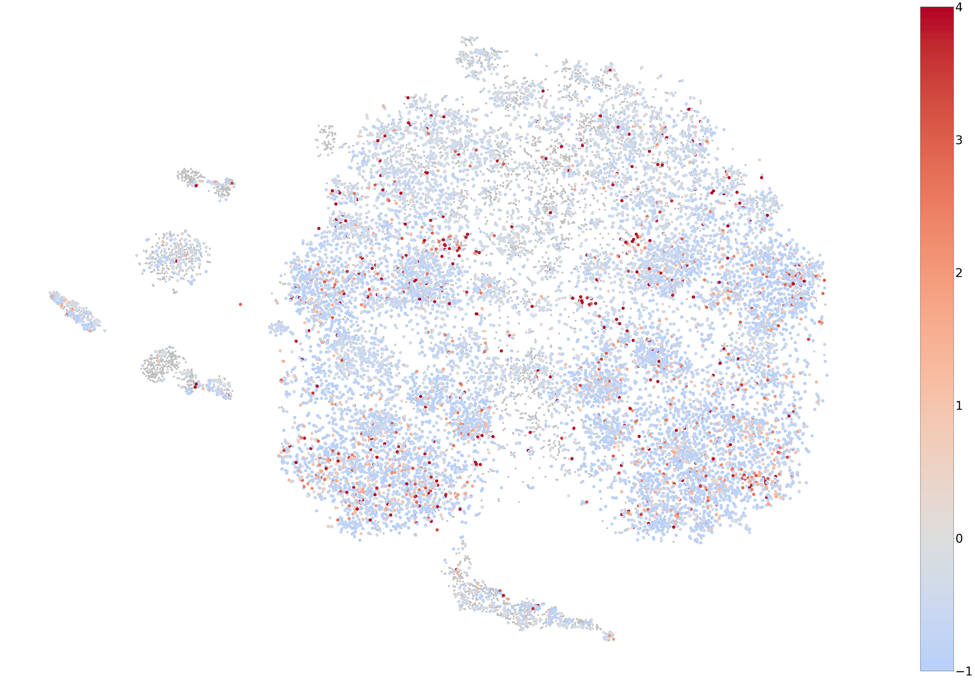


**Figure S13**: Gene co-expression map with genes colored by z-scores obtained using GTEx muscle expression data.


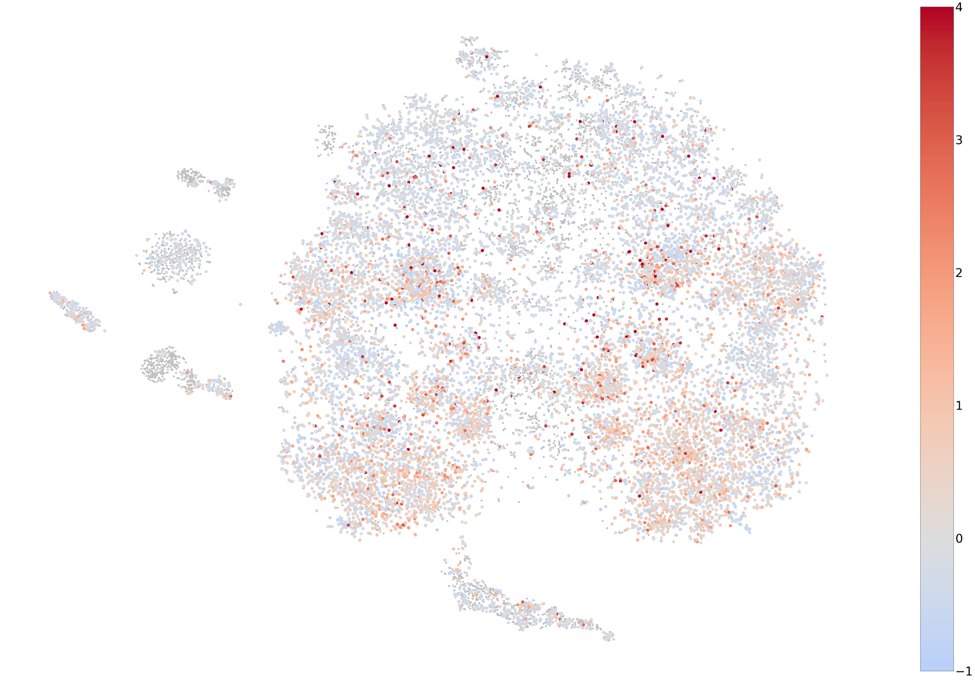


Nerve

**Figure S14**: Gene co-expression map with genes colored by z-scores obtained using GTEx nerve expression data.


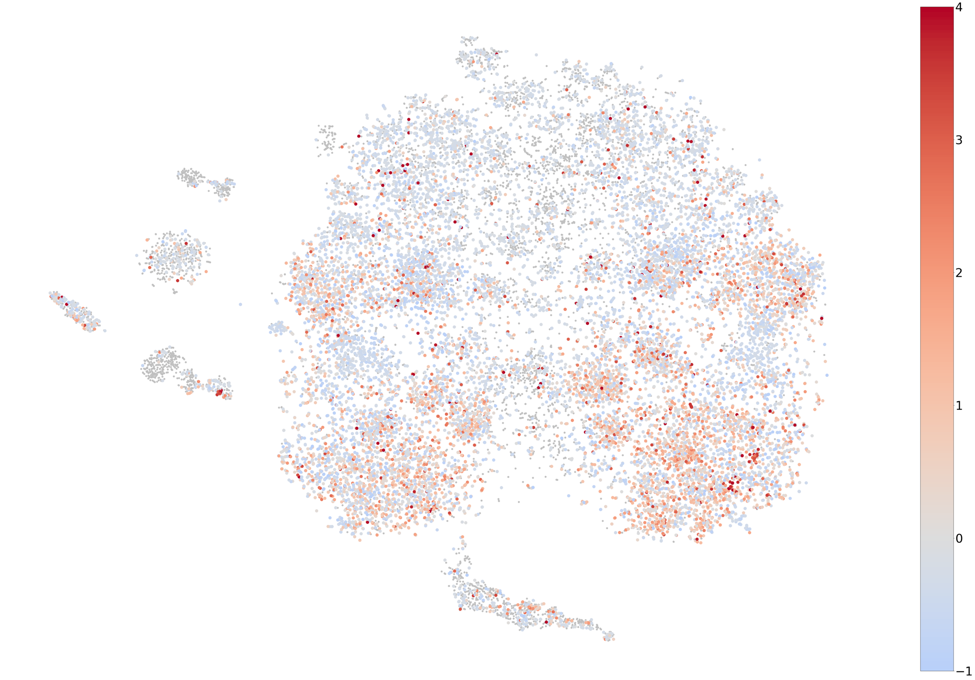


Ovary

**Figure S15**: Gene co-expression map with genes colored by z-scores obtained using GTEx ovary expression data.


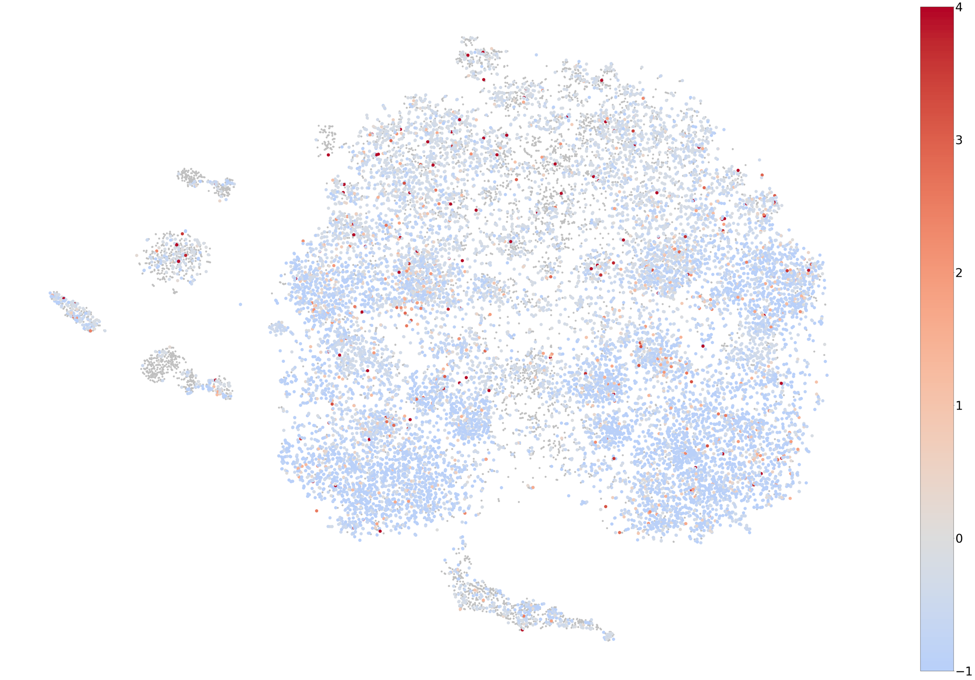


Pancreas

**Figure S16**: Gene co-expression map with genes colored by z-scores obtained using GTEx pancreas expression data.


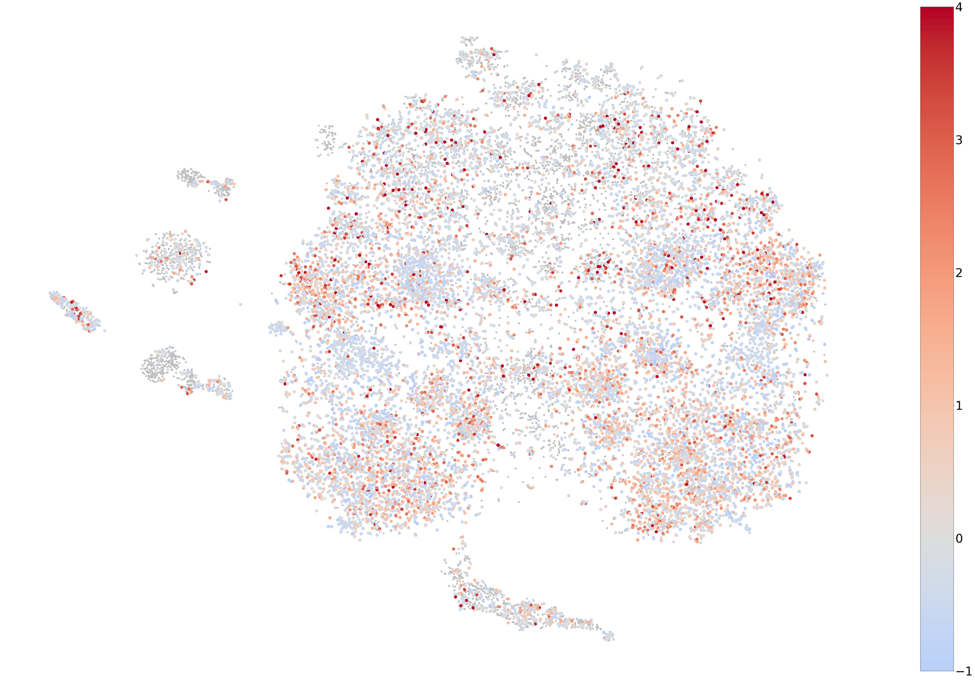


Pituitary

**Figure S17**: Gene co-expression map with genes colored by z-scores obtained using GTEx pituitary expression data.


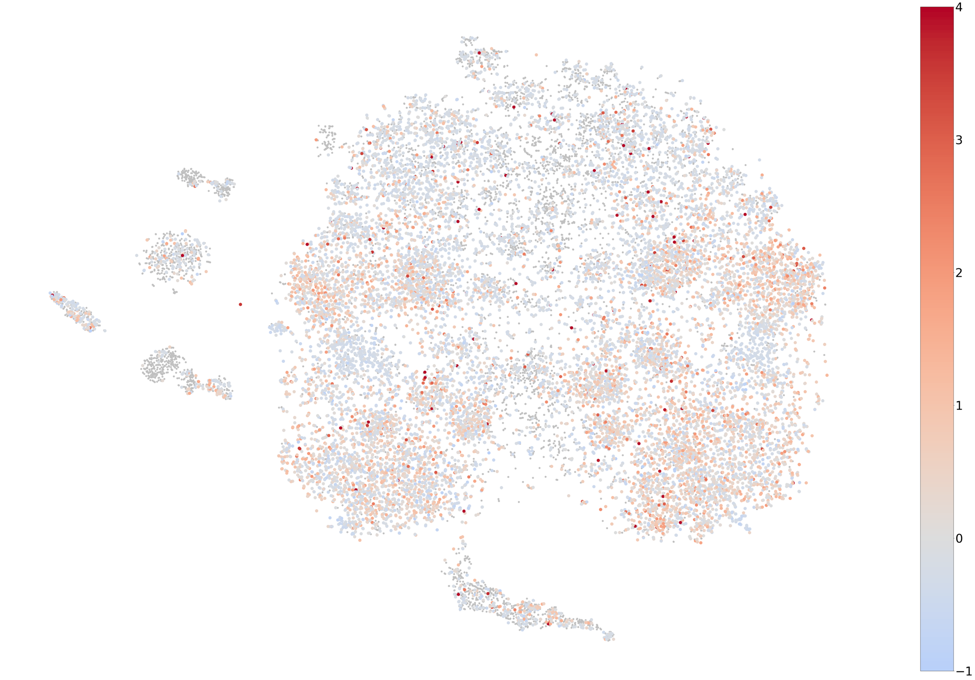


Prostate

**Figure S18**: Gene co-expression map with genes colored by z-scores obtained using GTEx prostate expression data.


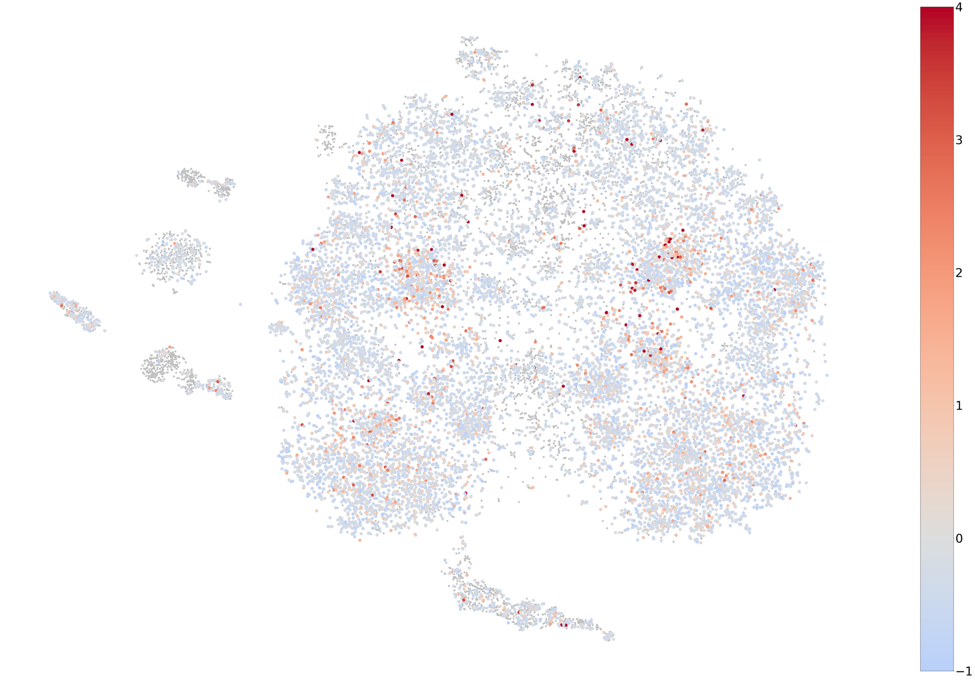


Salivary

**Figure S19**: Gene co-expression map with genes colored by z-scores obtained using GTEx salivary gland expression data.


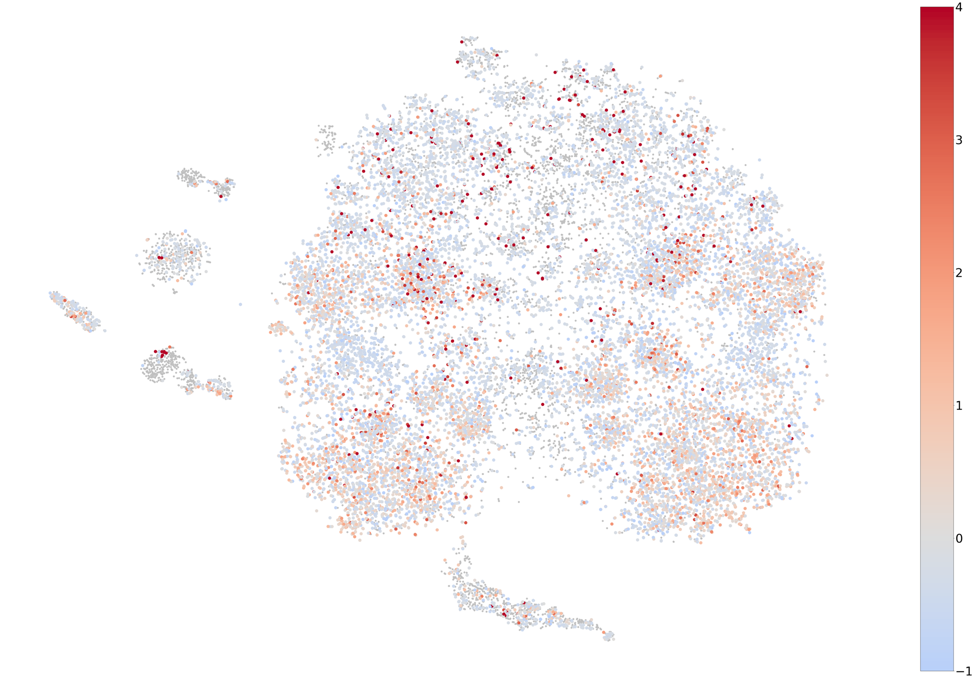


Skin

**Figure S20**: Gene co-expression map with genes colored by z-scores obtained using GTEx skin expression data.


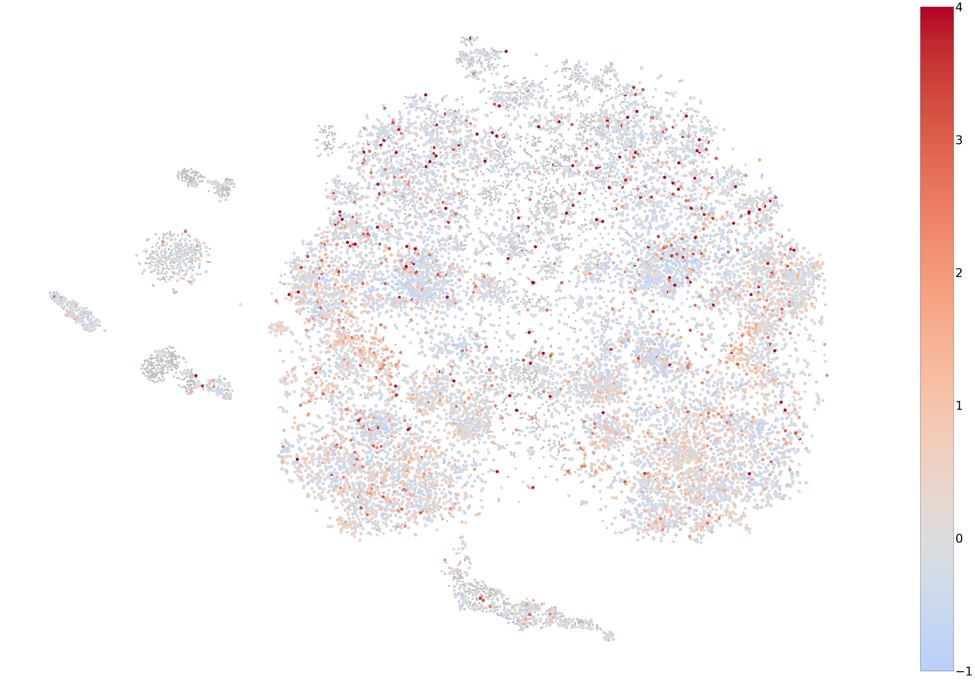


Small intestine

**Figure S21**: Gene co-expression map with genes colored by z-scores obtained using GTEx small intestine expression data.


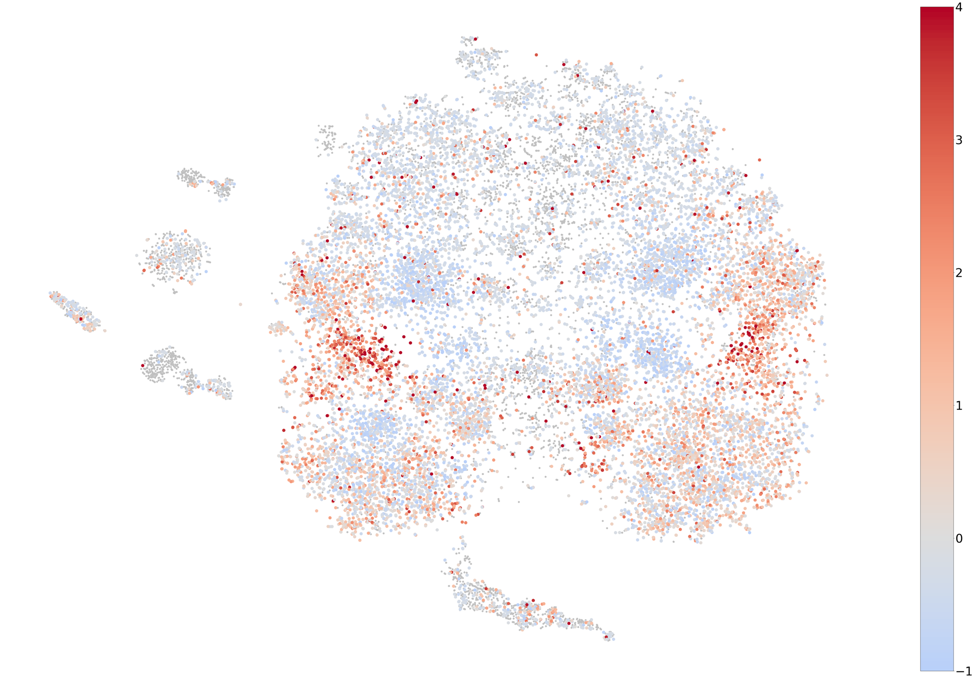


Spleen

**Figure S22**: Gene co-expression map with genes colored by z-scores obtained using GTEx spleen expression data.


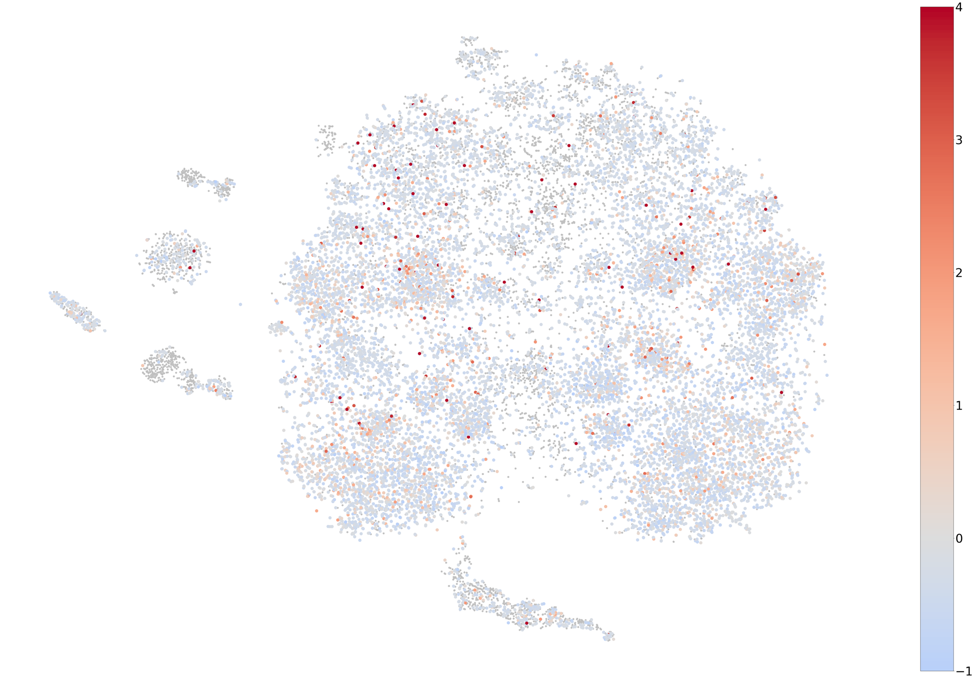


Stomach

**Figure S23**: Gene co-expression map with genes colored by z-scores obtained using GTEx stomach expression data.


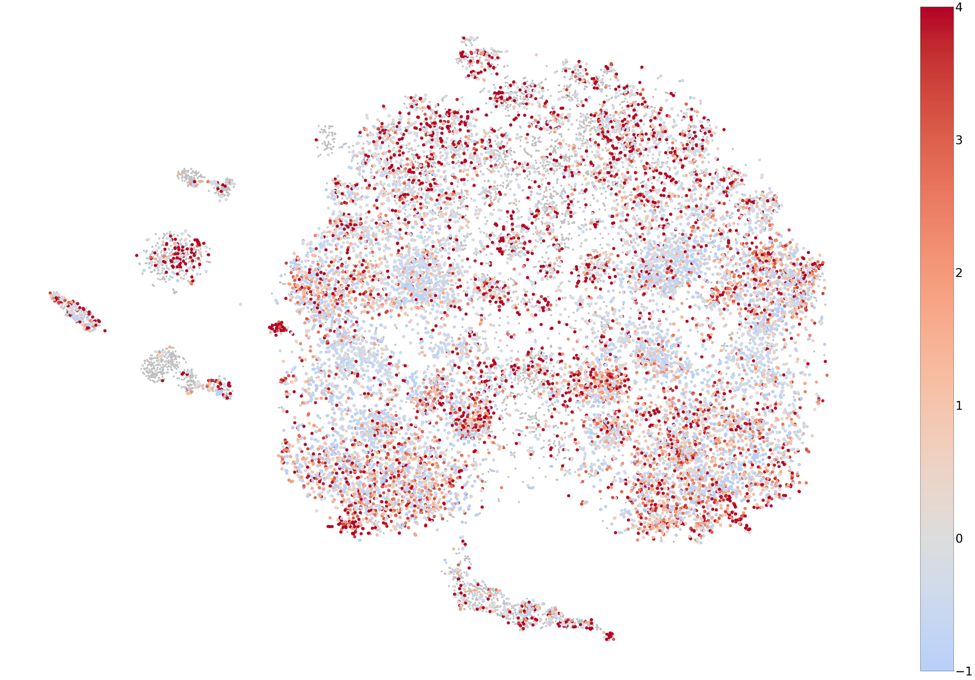


Testis

**Figure S24**: Gene co-expression map with genes colored by z-scores obtained using GTEx testis expression data.


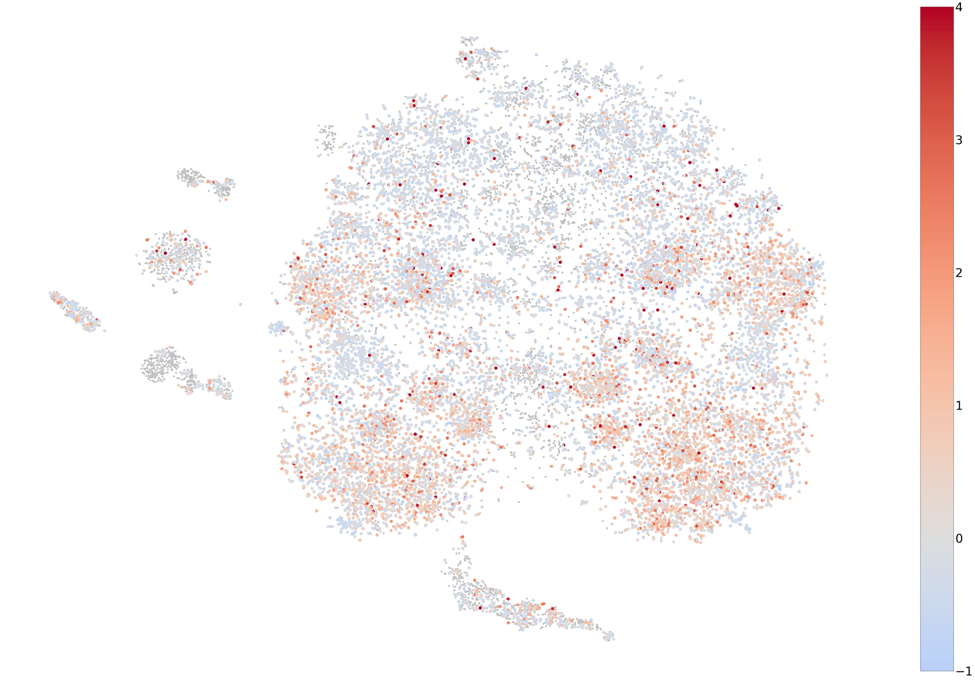


Thyroid

**Figure S25**: Gene co-expression map with genes colored by z-scores obtained using GTEx thyroid expression data.


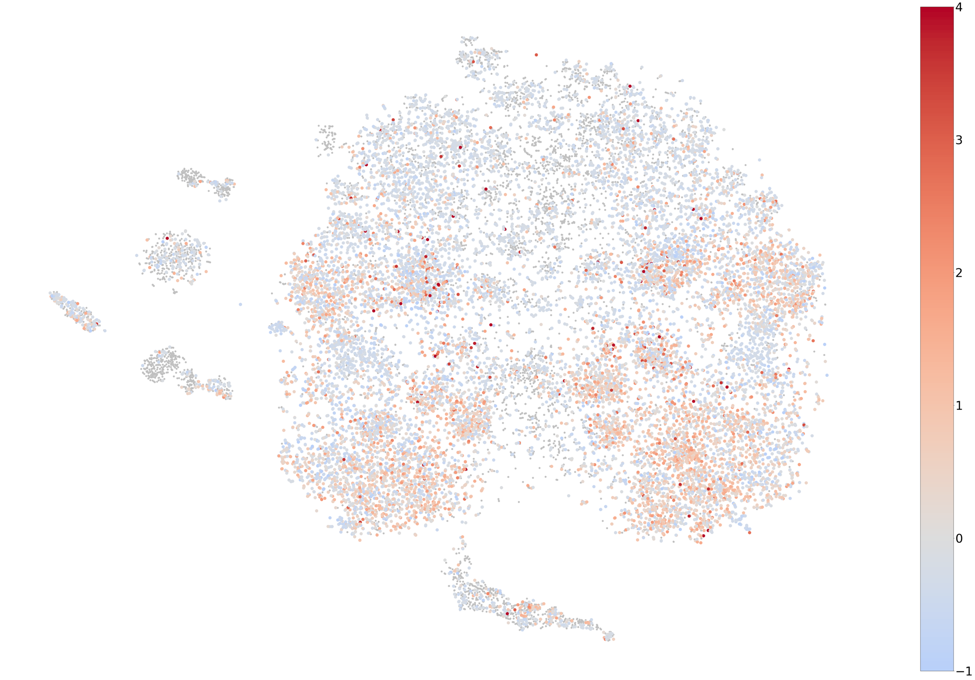


Uterus

**Figure S26**: Gene co-expression map with genes colored by z-scores obtained using GTEx uterus expression data.


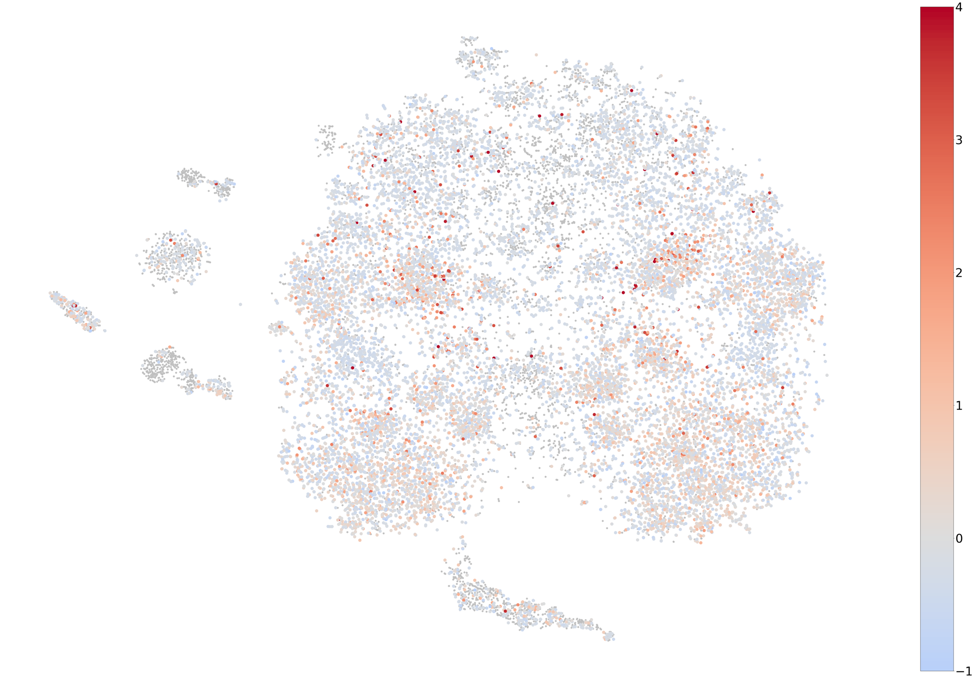


Vagina

**Figure S27**: Gene co-expression map with genes colored by z-scores obtained using GTEx vagina expression data.
